# *N*-Butyl-2-Cyanoacrylate-Assisted Balloon-Occluded Retrograde Transvenous Obliteration (NARTO) of Gastric Varices and Splenorenal Shunts

**DOI:** 10.1007/s00270-025-04137-0

**Published:** 2025-08-04

**Authors:** Eisuke Shibata, Hidemasa Takao, Osamu Abe

**Affiliations:** Department of Radiology, Graduate School of Medicine, The University of Tokyo, 7-3-1 Hongo, Bunkyo-Ku, Tokyo, 113-8655 Japan

**Keywords:** Balloon-occluded retrograde transvenous obliteration, Embolization, Gastric varices, *n*-butyl-2-cyanoacrylate, Splenorenal shunts

## Abstract

**Purpose:**

This retrospective study investigated the efficacy of *n*-butyl-2-cyanoacrylate (nBCA)-assisted balloon-occluded retrograde transvenous obliteration (NARTO) of gastric varices and splenorenal shunts.

**Materials and Methods:**

Between November 2021 and February 2024, 16 patients (3 females, 13 males) underwent NARTO, consisting of balloon-occluded retrograde transvenous obliteration (BRTO) followed by nBCA embolization of the efferent draining vein without the balloon catheter remaining indwelling afterward, to treat gastric varices in 12 patients with liver cirrhosis or to improve portal vein flow or hepatic encephalopathy in four patients with splenorenal shunts after liver transplantation.

**Results:**

NARTO was technically successful in all 16 patients without major procedure-related complications. There was no migration of nBCA nor adhesion of nBCA to the balloon catheter. Follow-up contrast-enhanced CT showed no recurrence of gastric varices. For splenorenal shunts after liver transplantation, portal steal syndrome and hepatic encephalopathy improved after NARTO.

**Conclusion:**

NARTO is a simple and efficient technique to occlude gastric varices and portosystemic shunts, avoiding the need for the indwelling of a balloon catheter after injecting the sclerosant.

**Graphical Abstract:**

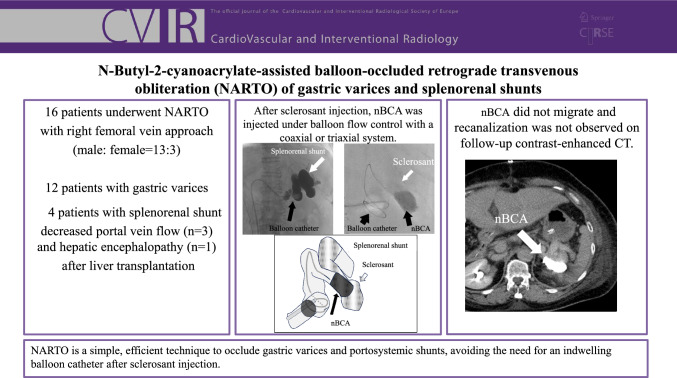

## Introduction

Gastric varices are known as dilated portosystemic collateral pathway and are found in up to 20% of patients with liver cirrhosis [1, 2]. Bleeding from gastric varices is more serious than bleeding from esophageal varices, with a higher mortality rate. Spontaneous portosystemic shunts (SPSs) are found in 20–35% of candidates for liver transplantation [3–6]. SPSs may be associated with postoperative liver failure due to portal steal syndrome, because portal inflow to the transplanted liver graft depends on the patency of the portal venous system, the diameter of the portal vein, and the presence of an SPS [7, 8].The left renal vein is often ligated to prevent portal steal syndrome due to an SPS and graft failure [9, 10].

Balloon-occluded retrograde transvenous obliteration (BRTO) is widely recognized as an endovascular treatment for gastric varices that is less invasive than surgery [11]. However, in conventional BRTO, the indwelling of a balloon catheter is required to obtain the effect of sclerosing agents. To overcome the disadvantages or complications of a indwelling balloon catheter in conventional BRTO, several modified BRTO procedures have been reported, such as plug-assisted retrograde transvenous obliteration (PARTO), coil-assisted retrograde transvenous obliteration (CARTO), and CARTO-II [12–14]. The use of nBCA in BRTO has only been described in case reports [15, 16]. We previously reported the technique of n-butyl-2-cyanoacrylate-assisted balloon-occluded retrograde transvenous obliteration (NARTO) for gastric varices [16]. This technical note aims to provide additional confirming data on NARTO in patients with gastric varices or splenorenal shunts.

## Materials and Methods

The institutional review board approved this retrospective study and waived the requirement for informed patient consent.

### Patients

Between November 2021 and February 2024, 16 patients (mean ± standard deviation age, 57.5 ± 14 years; range, 32–72 years; 3 females and 13 males) underwent NARTO (Table 1). Twelve of these patients underwent NARTO for gastric varices associated with liver cirrhosis. Of these 12 patients, three were included in our previously published case report [16]. Five patients experienced bleeding from gastric varices and two of them underwent endoscopic injection sclerotherapy before NARTO. The other seven patients had a high risk of bleeding from gastric varices. The remaining four patients underwent NARTO to treat splenorenal shunts after living-donor liver transplantation for insufficient portal vein flow (*n* = 3) or hepatic encephalopathy (*n* = 1). After discussion with gastroenterologists and surgeons, endovascular therapy was planned.

**Table 1:** Characteristics of individual patients

Patient no	Age	Sex	Treatment target	Underlying disease
1	60	M	Splenorenal shunt	Liver transplantation due to NASH
2	44	M	Gastric varices	Alcohol- and hepatitis B virus-related liver cirrhosis
3	62	M	Gastric varices	Liver cirrhosis due to NASH
4	64	F	Splenorenal shunt	Liver transplantation due to primary biliary cirrhosis
5	66	M	Gastric varices	HCV-related liver cirrhosis
6	78	F	Gastric varices	Primary biliary cirrhosis
7	50	M	Gastric varices	Alcohol-related liver cirrhosis
8	44	M	Gastric varices	Liver cirrhosis due to NASH
9	48	M	Gastric varices	Alcohol-related liver cirrhosis
10	32	M	Splenorenal shunt	Liver transplantation due to congenital biliary atresia
11	42	M	Splenorenal shunt	Liver transplantation due to alcohol-related liver cirrhosis
12	76	M	Gastric varices	Alcohol-related liver cirrhosis
13	50	F	Gastric varices	Overlapping autoimmune hepatitis and primary biliary cirrhosis
14	60	M	Gastric varices	HCV-related liver cirrhosis
15	72	M	Gastric varices	Alcohol-related liver cirrhosis
16	72	M	Gastric varices	Liver cirrhosis due to NASH

*HCV* hepatitis C virus,* F* female, *M* male, *NASH* nonalcoholic steatohepatitis

### NARTO Procedure

NARTO consisted of the balloon-assisted infusion of a sclerosant into the gastric varices or the shunt (BRTO), followed by nBCA embolization of the efferent draining vein under balloon flow control without the balloon catheter remaining indwelling afterward. All the procedures were mainly performed by two experienced interventionalists with 20 and 12 years of experience. All endovascular treatments were performed using a right femoral vein approach under local anesthesia. In 13 patients, an 8 Fr sheath was manually shaped into a reversed S shape using heat, and directly inserted into the shunt from the right femoral vein. A 6 Fr (Selecon MP catheter II, Terumo Clinical Supply, Gifu, Japan; balloon diameter: 20 mm), a 6 Fr (Nipro occlusion catheter, Nipro, Osaka, Japan; balloon diameter: 30 mm), or a 5.2 Fr (Selecon MP catheter II, Terumo Clinical Supply; balloon diameter 9 mm) balloon catheter was used for flow control. In the other three patients, a 10 Fr sheath (Medikit, Tokyo, Japan) was used to insert a 9 Fr and 5 Fr double-balloon coaxial catheter system for BRTO (Candis, Medikit; balloon diameters: 20 and 10 mm).

The balloon catheter was advanced retrogradely far enough to occlude the efferent draining vein. After confirming that the blood flow of the draining vein was completely blocked with the balloon and that either the gastric varices or the shunt was shown by balloon-occluded retrograde transvenous venography, 5% ethanolamine oleate iopamidol (EOI), a mixture of 10% monoethanolamine oleate (Oldamin, ASKA Pharmaceutical, Osaka, Japan), and the same volume of nonionic contrast media were injected under balloon flow control, followed by a waiting time of typically 15 min. For renal protection, 4000 U of haptoglobin was intravenously infused, starting before sclerosant injection.

To embolize the draining vein after sclerosant injection, nBCA (Histoacryl, B. Braun, Melsungen, Germany) (mainly 33% [nBCA:lipiodol = 1:2]) was infused through a coaxial or a triaxial microcatheter system after injecting 5% glucose under balloon flow control in all patients (Table 2). After nBCA injection, we waited for several minutes (typically 2 min) to allow nBCA to polymerize. In 14 patients, we only injected nBCA into the proximal space where the sclerosant was injected. In the other two patients, we used metallic coils before injecting nBCA. In the first case of NARTO (patient no.16), nBCA was used to embolize the draining vein completely after placement of metallic coils. In the other case (patient no.8), metallic coils were used to assist and control the distribution of the sclerosant and nBCA.

**Table 2:** Embolization procedure and outcomes of individual patients

Patient no	Agents used to embolize the shunts (nBCA %^a^)	Embolization of collateral veins	Microcatheter system	Complications	Time of last follow-up CT (days)
1	Monoethanolamine oleate, nBCA (33%)	−	Triaxial	−	151
2	Monoethanolamine oleate, nBCA (33%)	+	Triaxial	−	−
3	Monoethanolamine oleate, nBCA (33%)	−	Triaxial	−	232
4	Monoethanolamine oleate, nBCA (33%)	−	Triaxial	−	66
5	Monoethanolamine oleate, nBCA (33%)	+	Triaxial	−	256
6	Monoethanolamine oleate, nBCA (33%)	−	Coaxial	−	367
7	Monoethanolamine oleate, nBCA (50%)	−	Triaxial	−	388
8	Monoethanolamine oleate, metallic coils, nBCA (50%)	−	Triaxial	−	396
9	Monoethanolamine oleate, nBCA (50%)	−	Triaxial	−	398
10	Monoethanolamine oleate, nBCA (50%)	−	Triaxial	−	356
11	Monoethanolamine oleate, nBCA (50%)	−	Triaxial	−	481
12	Monoethanolamine oleate, nBCA (33%)	+	Coaxial	−	597
13	Monoethanolamine oleate, nBCA (33%)	+	Coaxial	−	597
14	Monoethanolamine oleate, nBCA (33%)	−	Triaxial	−	798
15	Monoethanolamine oleate, nBCA (33%)	−	Coaxial	−	566
16	Monoethanolamine oleate, metallic coils, nBCA (25%)	−	Coaxial	−	874

^a^Concentration of nBCA diluted with lipiodol

*nBCA n*-butyl-2-cyanoacrylate

## Results

NARTO was technically successful in all 16 patients. There were no major procedure-related complications. Although three patients experienced fever and one had pain (Grade 1a in the modified CIRSE classification system for complications’ reporting [17]) after treatment, these symptoms resolved with conservative treatment. Technically, nBCA migration did not occurred during procedures. Furthermore, nBCA did not adhere to the balloon catheters.

The mean ± standard deviation follow-up with contrast-enhanced CT was 435 ± 216 days (range, 66–874 days) in all patients, except for a patient (patient no. 2) who had not undergone follow-up CT. The follow-up contrast-enhanced CT images of 12 patients with gastric varices showed that all the gastric varices were thrombosed. None of the patients with gastric varices needed further treatment, and none of the patients had bleeding from gastric varices. Of the four patients with splenorenal shunts after liver transplantation, ultrasonography revealed that portal vein flow improved after NARTO in three patients with portal steal syndrome, and the other patient showed moderate improvement in hepatic encephalopathy and blood ammonia levels. None of the patients required further treatment of splenorenal shunts.

**Fig. 1:**
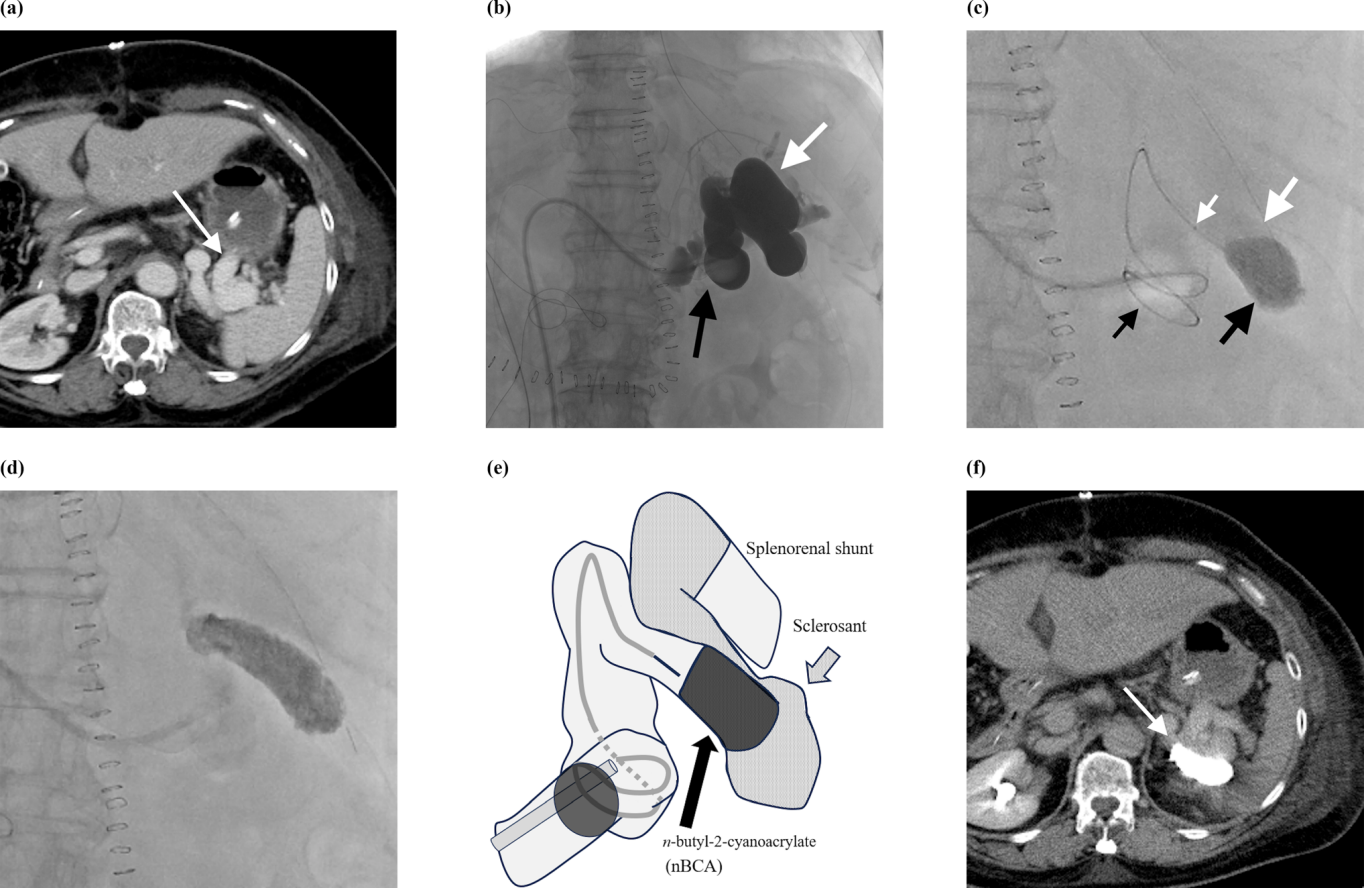
A 64-year-old female with a splenorenal shunt after liver transplantation (patient no. 4). **a** Contrast-enhanced CT image shows a splenorenal shunt (arrow), which caused a decrease in the portal flow to the liver graft. **b** After inserting a balloon catheter (black arrow) into the shunt via a right femoral vein approach, balloon-occluded retrograde venography revealed the whole shunt and stagnation of blood flow in the shunt (white arrow). **c** After 5% ethanolamine oleate iopamidol injection (large white arrow), 33% *n*-butyl-2-cyanoacrylate (nBCA; large black arrow) was injected with a triaxial microcatheter system (a 1.9Fr microcatheter and a 2.7Fr microcatheter; small white arrow) under balloon flow control (small black arrow). **d** The injected nBCA formed a large cluster in the shunt and was stable. **e** The scheme shows n-butyl cyanoacrylate-associated retrograde transvenous obliteration for a splenorenal shunt with a triaxial system under balloon flow control. **f** Follow-up contrast-enhanced CT performed 66 days after embolization shows the nBCA cluster in the shunt (arrow), consistent with the angiographic findings, without nBCA migration

An illustrative case demonstrating the treatment of a splenorenal shunt is shown in Fig. 1.

## Discussion

NARTO is a simple technique that uses nBCA to occlude portosystemic shunts, such as gastric varices and splenorenal shunts, and avoids the need for a balloon catheter to remain indwelling after sclerosant injection. Although BRTO is effective for gastric varices, modified BRTO procedures have been proposed to overcome the disadvantages of the original BRTO. For example, Gwon et al. reported PARTO using a vascular plug and Gelfoam slurry without an indwelling balloon or sclerosant [12]. Lee et al. introduced CARTO using coils and Gelfoam slurry without limitation on the vascular plug [13]. Subsequently, Kim et al. reported CARTO-II, in which coils were placed after initial BRTO with sclerosants [14]. However, the average procedure time for CARTO and CARTO-II is longer, and these procedures may be more expensive than PARTO, due to the need to insert multiple coils.

There are several advantages of using nBCA in NARTO. The first advantage is that nBCA can be used for embolization regardless of the vessel size, once blood flow is controlled with a balloon catheter.

The second advantage of using nBCA is that it is quicker to inject nBCA than to insert a sufficient number of coils to embolize the draining vein. When inserting metallic coils into large varices or shunts, many coils may be needed to achieve complete occlusion, which may increase the cost of the procedure. In nBCA embolization, a single injection of nBCA is usually sufficient to achieve complete occlusion.

The third advantage of using nBCA is that it is unnecessary to keep the indwelling balloon for a long time (i.e., overnight), unlike in standard BRTO. Additionally, it is easier to evaluate the treated area on follow-up contrast-enhanced CT using nBCA because it generates of fewer artifacts than coils.

The potential disadvantages of NARTO are related to possible complications associated with nBCA. The first complication is that nBCA may adhere to the catheters, which could lead to catheter fixation. Careful positioning of the microcatheter during nBCA injection is important to prevent it from adhering to the microcatheter or the balloon catheter. For safety reasons, we often use a triaxial microcatheter system to reduce the risk of nBCA adhering to larger catheters.

Another nBCA-related complication is the risk of nBCA migration after balloon deflation. This complication is similar to the risk of coil migration when using metallic coils in BRTO. We believe that injecting a sufficient volume of nBCA and waiting for an appropriate time are important to ensure the nBCA forms a large cluster and to allow nBCA to harden and adhere to the vascular walls [18].

There are several limitations in this study. First, this study included a small number of patients and it was performed at a single center. Larger studies are needed to confirm the effectiveness and safety of this procedure. Second, direct comparisons with other techniques, such as the original BRTO, PARTO, CARTO, and CARTO-II procedures, are needed to establish the usefulness of NARTO.

In conclusion, NARTO is a simple, efficient technique to occlude gastric varices and portosystemic shunts, avoiding the need for the indwelling of a balloon catheter after sclerosant injection.
